# Supplementary material for: Development and prognostic validation of a three-level NHG-like deep learning-based model for histological grading of breast cancer
Source: Breast Cancer Res. 2024 Jan 29;26:17. doi: 10.1186/s13058-024-01770-4 (PMC10823657; doi:10.1186/s13058-024-01770-4)
Supplement: Supplementary file 1 — Additional file 1. Table1: Baseline characteristics of the patients in SöS-BC-4 training set and the SCANB cohort. Figure 1: Boxplots of the distribution of number of tiles for each WSI in the two study cohorts. Table 2: Visualization of tiles from the SöS-BC4 training set. [file 13058_2024_1770_MOESM1_ESM.docx]

**Development and prognostic validation of a three-level NHG-like deep learning-based model for histological grading of breast cancer**

Abhinav Sharma, Philippe Weitz, Yinxi Wang, Bojing Liu, Johan Vallon-Christersson, Johan Hartman, Mattias Rantalainen

**Table of Contents:**

**Table1:** Baseline characteristics of the patients in SöS-BC-4 training set and the SCANB cohort.

**Figure 1:** Boxplots of the distribution of number of tiles for each WSI in the two study cohorts.

**Table 2:** Visualization of tiles from the SöS-BC4 training set.

**Table1:** Baseline characteristics of the patients in SöS-BC-4 training set and the SCANB cohort.

|  | **level** | **SöS-BC-4 cohort**  **(Training set)** | **SCANB cohort** |
| --- | --- | --- | --- |
| **n** |  | 1695 | 1262 |
| **Age (mean (SD))** |  | 61.52 (11.41) | 64.85 (12.27) |
| **ER status (%)** | **"negative"** | 159 (9.4) | 133 (10.5) |
|  | **"positive"** | 1413 (83.4) | 1119 (88.7) |
|  | **“NA”** | 123 (7.3) | 10 (0.8) |
| **Tumor size (%)** | **"(<20mm)"** | 892 (52.6) | 879 (69.7) |
|  | **"(>=20mm)"** | 407 (24.0) | 364 (28.8) |
|  | **“NA”** | 396 (23.4) | 19 (1.5) |
| **Lymph node (%)** | **"0"** | 1048 (61.8) | 884 (70.0) |
|  | **"1"** | 253 (14.9) | 344 (27.3) |
|  | **“NA”** | 394 (23.2) | 34 (2.7) |
| **HER2 status (%)** | **"negative"** | 1353 (79.8) | 1133 (89.8) |
|  | **"positive"** | 160 (9.4) | 103 (8.2) |
|  | **“NA”** | 182 (10.7) | 26 (2.1) |
| **RFS (mean (SD))** |  | 1745.14 (679.21) | 1974.42 (861.77) |
| **RFS_status (%)** | **"0"** | 1374 (81.1) | 1133 (89.8) |
|  | **"1"** | 110 (6.5) | 129 (10.2) |
|  | **“NA”** | 211 (12.4) |  |
| **clinicalNHG (%)** | **"NHG1"** | 357 (21.1) | 280 (22.2) |
|  | **"NHG2"** | 793 (46.8) | 608 (48.2) |
|  | **"NHG3"** | 545 (32.2) | 374 (29.6) |

**Figure 1:** Boxplots of the distribution of number of tiles for each WSI in the two study cohorts.


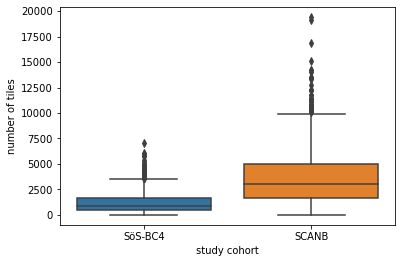


After the WSI preprocessing steps, invasive cancer tiles for each WSI were used for the model optimisation and validation. The difference in distribution of number of tiles per WSI for SöS-BC4 and SCANB cohort is mainly due to the difference in overlapping used between the two consecutive tiles in the WSI preprocessing step (Figure 1). No-overlap between the consecutive tiles was used in the SöS-BC4 cohort whereas an overlap of 50% between the two consecutive tiles was used in the SCANB cohort preprocessing (1).

**Table 2:** Visualization of tiles from the SöS-BC4 training set. Example tiles for predGrade 1,2 and 3 WSIs from the 5-fold CV test sets in SöS-BC4 training set. We selected 16 correctly classified predGrade WSIs, that were randomly sampled from the >=0.95, (>=0.40 & <=0.60) and <=0.10 slide-level scores for predGrade3, 2, and 1 WSIs respectively. Further, we randomly selected one tile from the top 5-percentile attention score for each WSI. Each row represents a unique WSI with four tiles as the column

| **predGrade3** |
| --- |
| 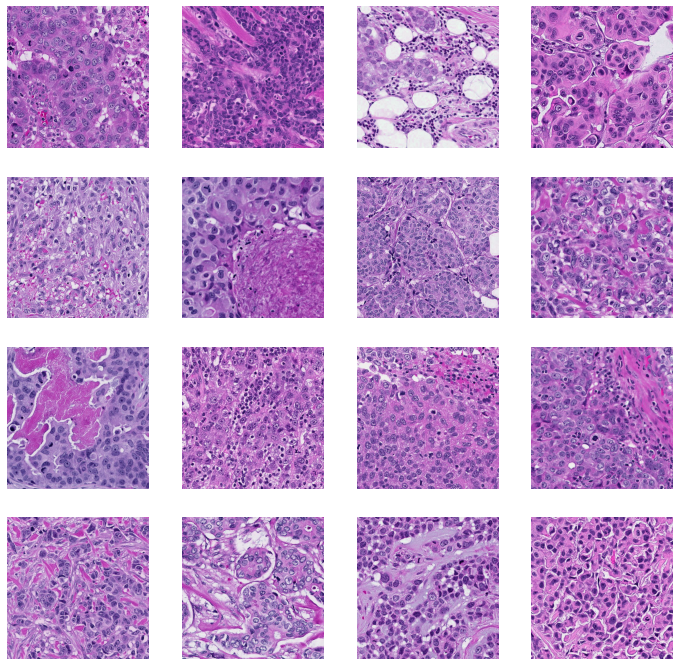 |
| **predGrade2** |
| 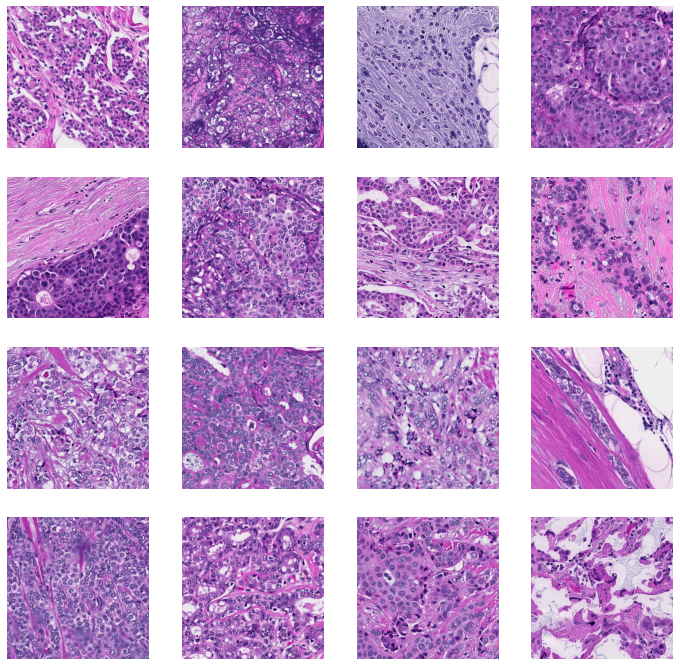 |
| **predGrade1** |
| **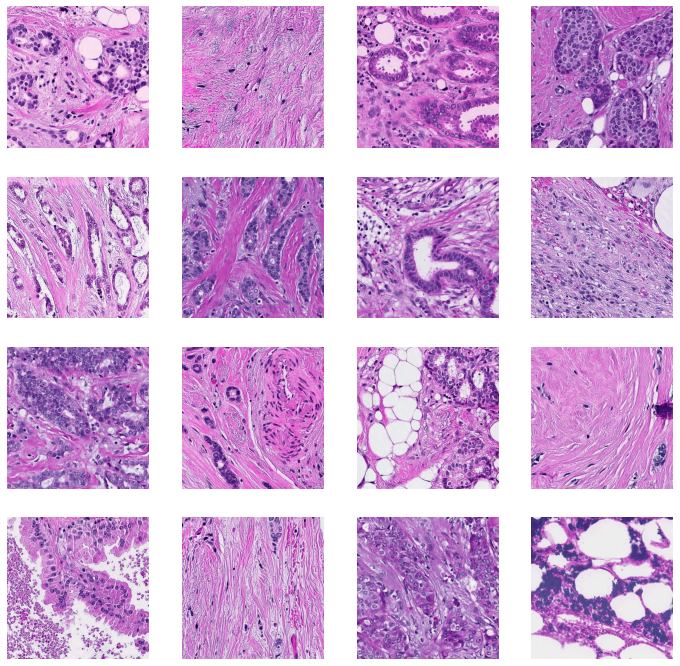** |

**References:**

1. Wang Y, Acs B, Robertson S, Liu B, Solorzano L, Wählby C, et al. Improved breast cancer histological grading using deep learning. Ann Oncol. 2022 Jan;33(1):89–98.
